# Higher dose of rivaroxaban for the treatment of venous thromboembolism in a 15-year-old Asian child with antithrombin deficiency: A case report and literature review

**DOI:** 10.1097/MD.0000000000041629

**Published:** 2025-02-28

**Authors:** Chun-Su Liang, Yue-Xin Chen, Fang Liu, Yue-Dong Yue, Li-Ping Du

**Affiliations:** a Department of Pharmacy, Peking Union Medical College Hospital, Chinese Academy of Medical Sciences & Peking Union Medical College, Beijing, China; b Department of Vascular Surgery, Peking Union Medical College Hospital, Chinese Academy of Medical Sciences & Peking Union Medical College, Beijing, China; c Department of Pharmacy, Beijing Luhe Hospital, Capital Medical University, Beijing, China; d Department of Pharmacy, Liaocheng People’s Hospital, Liaocheng, China.

**Keywords:** antithrombin deficiency, Asian child, rivaroxaban, venous thromboembolism

## Abstract

**Rationale:**

Deficiency in antithrombin (AT) can significantly increase the risk of venous thromboembolism (VTE). However, there is insufficient data on the efficacy and safety of anticoagulants in patients with AT deficiency, especially in children. In addition, Asian populations typically require a lower dose of rivaroxaban, and this may be even more pronounced in Asian children. This case aims to explore the potential efficacy and safety of a higher dose of rivaroxaban in an Asian child with AT deficiency and VTE.

**Patient concerns:**

A 15-year-old boy was referred to our center with severe deep vein thrombosis progression. The primary concern was the effective management of the thromboembolic events while minimizing the risk of bleeding, given the patient’s young age and AT deficiency.

**Diagnoses:**

The patient was diagnosed with pulmonary embolism and deep vein thrombosis with AT deficiency.

**Interventions:**

The patient was treated with a higher dose of rivaroxaban, which was 15 mg twice a day for 3 weeks, followed by 20 mg per day for 6 months, which was a relatively high dose for an Asian child.

**Outcomes:**

During the follow-up period, the patient did not experience any VTE events or bleeding events.

**Lessons:**

This case provides additional data on the efficacy and safety of direct oral factor Xa inhibitors in patients with VTE and AT deficiency. It suggests that for Asian children with AT deficiency, considering a higher dose of rivaroxaban could be beneficial, especially when the children’s height, weight, and age are close to adults.

## 1. Introduction

Venous thromboembolism (VTE), comprising deep vein thrombosis (DVT), and pulmonary embolism (PE), which affects nearly 10 million people every year worldwide.^[1]^ The annual incidence of acute VTE is 0.1% to 0.2%,^[1]^ while approximately 20% of individuals with VTE event die within 1 year (although often from the provoking condition).^[2]^ The clinical symptoms of DVT include leg pain, swelling, redness, localized tenderness on palpation, and prominent collateral superficial veins, while patients with pulmonary embolism present with breathlessness, pleuritic chest pain, hemoptysis, and tachycardia.^[1]^ Inherited antithrombin (AT) deficiency is a rare (0.02% to 0.2% in the general population) and often under-recognized medical condition that is associated with inadequate endogenous anticoagulation thought to result from impaired inhibition of serine protease coagulation factors.^[3]^ Typically, AT levels in patients with AT deficiency fell well below the normal range (approximately 80%–120%), with AT activity below 70% highly indicative of AT deficiency, while most patients with inherited, heterozygous AT deficiency exhibit AT activity levels in the range of 40% to 60%.^[4]^ Notably, patients with AT deficiency face a significantly increased risk of VTE, with the relative risk of first VTE 15 times higher than in the general population and the risk of recurrence is 4 times higher.^[5]^ Approximately 50% to 90% of patients with AT deficiency will develop VTE during their lifetime,^[6]^ with thrombotic events often manifesting between the age of 10 and 35 in 67% of patients with inherited AT deficiency.^[7]^

The recommended initial treatment for VTE involves the continuous administration of heparin. However, patients with AT deficiency may present with heparin resistance, which refers to the situation wherein patients require unusually high doses of heparin to achieve a therapeutic activated partial thromboplastin time (aPTT).^[8]^ The impact of low AT plasma levels on the anticoagulant effect of heparins, including low molecular weight heparin (LMWH), further complicates treatment. Direct oral factor Xa inhibitors, acting directly on the coagulation cascade independently of AT participation, offer the potential for a robust anticoagulant effect, even in patients with AT deficiency. However, there remains limited data in the use of rivaroxaban in AT deficiency and VTE patients, especially concerning dosage and duration in children. Evidences showed that the dosage of rivaroxaban used by Asian populations could be lower than that of other ethnic backgrounds. The Japanese (J)—EINSTEIN DVT and PE program^[9]^ less than the dosage in the global EINSTEIN DVT and PE study^[10,11]^ (Table 1). Besides, in the Japanese (J)—ROCKET AF study^[12]^ rivaroxaban-treated patients used the dosage of 15 mg once daily, lower than the dosage used in global ROCKET AF study^[13]^ (rivaroxaban at a daily dose of 20 mg). This case report presents a 15-year-old Chinese VTE patient with AT deficiency, who received the oral factor Xa inhibitor rivaroxaban. The adolescent patient received an adult dose of 15 mg twice a day for 3 weeks, followed by a reduced dose of 20 mg per day for 6 months. With this anticoagulation regimen, the patient achieved a good outcome without bleeding or other adverse reactions.

**Table 1:** Rivaroxaban dose for venous thromboembolism.

	Starting dose (for 3 wk)	Maintenance dose
Global adults (EINSTEIN trial)	15 mg twice daily	20 mg once daily
Asian adults (J-EINSTEIN trial)	10 mg/15 mg twice daily	15 mg once daily
Global children (EINSTEIN-Jr trial)	20 mg once daily (max)	20 mg once daily (max)
The Asian child in this case	15 mg twice daily	20 mg once daily

## 2. Case report

A 15-year-old boy, 174 cm/81.1 kg (BMI 26.8), was referred to our center with severe DVT progression. The patient had been admitted to another hospital a week prior due to swelling in the right lower limb, and was diagnosed by ultrasound with thrombosis in the right common iliac vein and external iliac vein. A retrievable inferior vena cava filter was inserted and urokinase was intravenously administered in addition to LMWH therapy. Subsequently, the patient’s condition became worse. After 1 week of treatment, contrast-enhanced computed tomography of chest, abdomen and pelvis indicated inferior vena cava thrombosis (visible both above and below the filter), portal vein thrombosis, and right iliac vascular thrombosis. Multisystem venous ultrasound revealed thrombosis in the inferior vena cava, bilateral common iliac vein, external iliac vein, and common femoral vein. Notably, the patient’s grandmother had a history of thrombosis in her 60 seconds but the details were unknown. Upon arrival at our hospital, his temperature was 36.0 °C, with a pulse of 103 bpm, blood pressure of 125/71 mm Hg, respiratory rate of 19 breaths per minute and oxygen saturation of 97%.

After admission, a computed tomographic pulmonary angiography and computed tomographic venography showed massive thrombi in the right pulmonary artery and bilateral deep vein thrombosis. Laboratory examinations showed a normal complete blood count and biochemistry profile. C-reactive protein and d-dimer levels were elevated (71.84 and 3.47 mg/L fibrinogen equivalent unit (FEU), respectively; normal range of d-dimer: 0–0.55 mg/L FEU, using immunoturbidimetry). Urokinase and LMWH were replaced with unfractionated heparin administered by continuous intravenous pumping, the target aPTT was set at 60 to 80 seconds (normal range: 23.3–32.55 s, using coagulation method), and the pumping rate was gradually titrated from the initial 18 U/kg/h to as high as 37 U/kg/h (~72,000 U/d) before the aPTT reached the target range. With such an unusually high dose of heparin (>35,000 U/d), heparin resistance^[8]^ was suspected and AT activity was tested for the patient. The results showed a markedly low value of AT activity (27%; normal range: 80%–120%, using chromogenic substrate method). Protein C and protein S plasma levels were within the normal range, and no lupus anticoagulant, anticardiolipin antibody or anti-β2GP1 antibody were detected.

**Figure 1.**
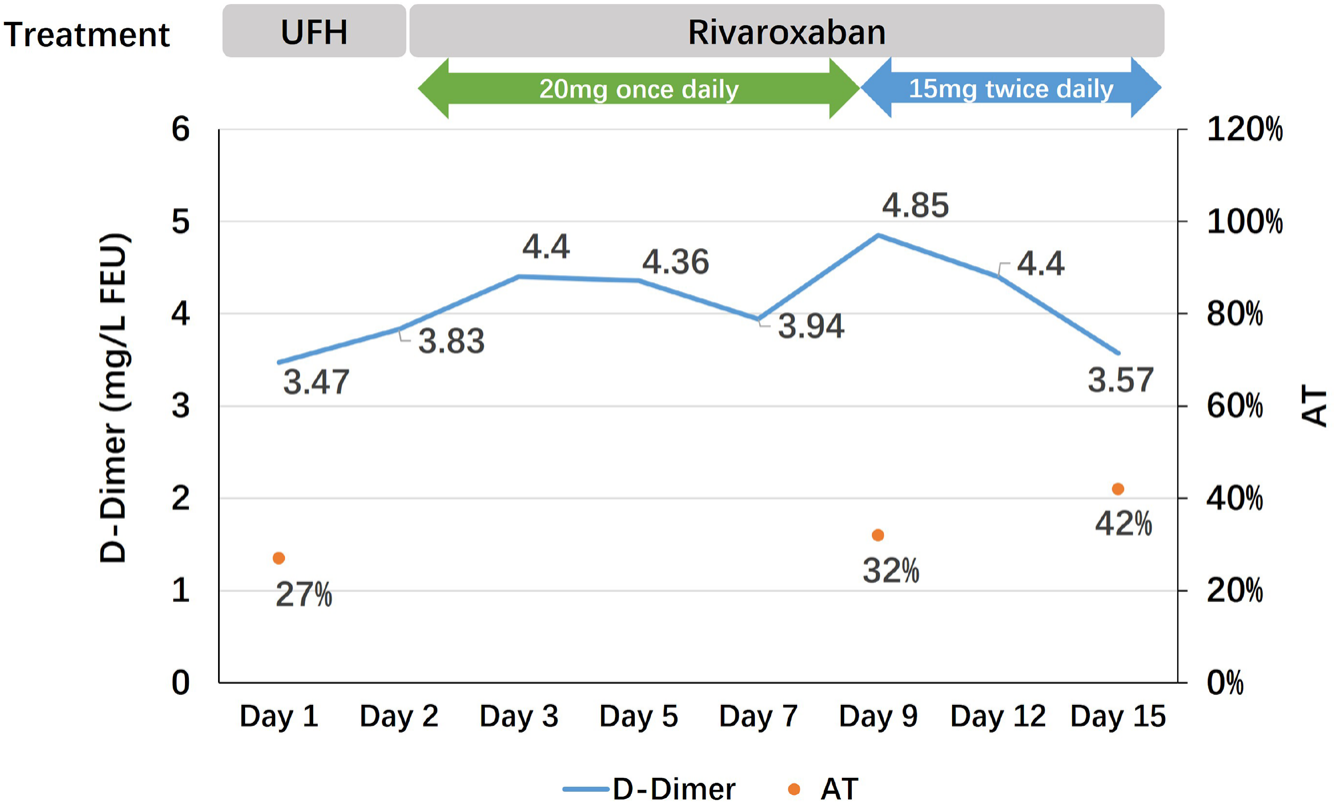
Changes in d-dimer and antithrombin activity during hospitalization. UFH: unfractionated heparin.

**Figure 2.**
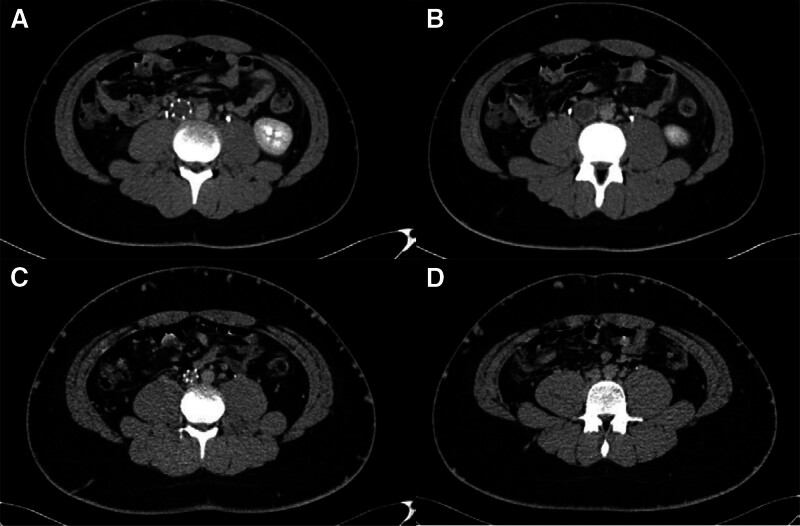
Comparison of computed tomographic venography (CTV) before and after medication. Figure (A) and (B) are before medication while (C) and (D) are after medication.

Based on findings from several examinations, the patient was diagnosed with PE and DVT with suspected AT deficiency. Because of the low AT activity, non-heparin anticoagulants became a priority. Based on the results of EINSTEIN-Jr study,^[14]^ rivaroxaban can be used for the treatment of acute VTE in children under 18 years of age. Therefore, rivaroxaban 20 mg daily was given to the patient based on his body weight, and d-dimer level was closely monitored to identify anticoagulant efficacy. After 1 week of rivaroxaban intake, the patient’s d-dimer was elevated from 3.83 to 4.85 mg/L FEU. Considering this boy’s age and weight close to that of an adult, the dose of rivaroxaban was adjusted to the acute thrombotic dose in adult (15 mg twice a day for 3 weeks, then reduced to 20 mg per day) after the informed consent of the patient and his parents. One week later, the patient reported no chest discomfort and the serum d-dimer level showed a significant decrease, and the AT activity was still lower than normal (42%) after heparin withdrawal (Fig. 1). Subsequently, the patient was discharged with continued treatment of rivaroxaban and regular follow-ups. Six months after discharge, the patient’s serum d-dimer was found to be normal (0.15 mg/L FEU), and repeat computed tomographic venography showed the thrombotic load was much less than before (Fig. 2). The patient had no bleeding complications occurred and was satisfied with the treatment he received.

## 3. Discussion

Anticoagulation serves as the fundamental treatment for VTE,^[15]^ and in patients with AT deficiency basically follows the standard management of venous thrombosis and inherited major thrombophilias.^[16]^ During the acute phase of VTE, especially in case of severe or recurrent thrombosis and/or heparin resistance, AT supplementation can be used with heparin.^[6,17]^ After the acute period, it is possible to switch to oral anticoagulation and antivitamin K treatments (vitamin K antagonists, VKAs) are preferred.^[16]^ However, there is a paucity of clinical evidence supporting the use of direct oral anticoagulants (DOACs) in patients with AT deficiency. Because DOACs act directly on the coagulation cascade without the participation of AT, these drugs might provide a potent anticoagulant effect even for patients with AT deficiency in theory. The potential utility of DOACs, especially direct oral factor Xa inhibitors, for the treatment of VTE in patients with AT deficiency still need more evidence.

The role of DOACs in treating inherited thrombophilia remains unclear, though some published data suggests their use may be effective.^[3]^ Recently, a prospective cohort study compared DOACs (cases) to heparin/VKAs (controls) for VTE treatment and secondary prevention in adult patients with inherited thrombophilia.^[18]^ This study, encompassing a large cohort of adult patients with acute VTE and inherited thrombophilia (n = 577, matched for age, sex, ethnicity, and thrombophilia type), of which 29 patients had AT deficiency, demonstrated DOACs’ efficacy comparable to that of heparin/VKAs. The efficacy and safety of rivaroxaban has also been demonstrated in the EINSTEIN study. Notably, the EINSTEIN trials^[10,11]^ included a small percentage of patients with thrombophilic disorders (5%–7%), with no specific information on the type of hypercoagulable disorder recorded. A literature review^[19]^ assessing the safety and efficacy of DOACs for VTE in select patients with hypercoagulable disorders showed that the safety and efficacy of a DOAC is highly dependent on the type of hypercoagulable disease state. Current trials showed that edoxaban, rivaroxaban, and apixaban are effective for treating cancer-associated thrombosis,^[19]^ with apixaban being the preferred choice due to lower bleeding rates compared to standard treatment. And a case report from Japan^[20]^ proved that edoxaban could be a useful treatment option for cancer patients with VTE and AT deficiency.

Rivaroxaban is a direct factor Xa inhibitor that can produce anticoagulant effect without the need for cofactors such as AT. To prevent a 30% decrease in anticoagulant activity, rivaroxaban at a dose of 15/20 mg should be consumed with food. A pilot study^[21]^ randomly assigned VTE patients with AT deficiency due to nephrotic syndrome into the rivaroxaban group (n = 8) and LMWH group (n = 8), and results showed that 7/8 patients achieved a primary endpoint (thrombus dissolution or a >90% decrease in thrombus volume in 4 weeks) in each of the 2 groups. Notably, at week 2 the patients whose AT levels and functional activity remained low in the LMWH group did not achieve the primary endpoint, which showed the potential benefits of rivaroxaban over LMWH treatment.

However, the efficacy and safety of rivaroxaban in patients with AT deficiency remain to be established, especially in children. In the EINSTEIN-Jr phase 3 trial,^[14]^ a total of 17 children patients exhibited deficiencies in AT, protein C, or protein S, with 15 of them being treated with rivaroxaban, and the dose was based on bodyweight (15 mg per day is the maximum daily dose for patients at a bodyweight 30–50 kg and 20 mg per day is the maximum daily dose for patients ≥50 kg).

Case reports have indicated the efficacy of rivaroxaban for the treatment of VTE in patients with AT deficiency (Table 2),^[22–32]^ and the dose of rivaroxaban was mostly 20/30 mg per day. But the maximum daily dose did not exceed 20 mg in both the 2 children cases.^[22,23]^ Evidences showed that Asian ethnicity could use smaller doses of rivaroxaban to achieve the same effect as other ethnic backgrounds using higher doses, and in special populations, low-dose rivaroxaban had shown better safety in Asians. In a retrospective study in Taiwanese,^[33]^ 10 mg once daily showed the same effect but better safety than 15 mg once daily in patients with renal insufficiency (eGFR < 50 mL/min per 1.73 m^2^). Thus, theoretically, the dosage of rivaroxaban used by Asian children should be even lower than that of other ethnic backgrounds.

**Table 2:** Case reports published venous thromboembolism patients with AT deficiency treated with rivaroxaban.

No.	Age (yr)	Gender	Indication	Dose	Outcome	Reference
1	11	M	Renal venous infarction, thrombosis, AT deficiency	10 mg twice daily for 1 d and 5 mg twice daily for 2 d and then 10 mg twice daily for 2 wk	No thrombosis and other side effects or complications occurred in the following 3 mo	^[22]^
2	12	F	Severe VTE, AT Budapest III mutation	20 mg once daily and then stopped after 5 d when the international normalized ratio was stable >2.0	On follow-up 3 mo later, the patient was doing clinically well	^[23]^
3	19	M	Multiple recurrences of VTE, AT deficiency	15 mg twice daily for 3 wk and then 15 mg per day continued for 6 mo	No recurrence of PTE or DVT during the 10-mo follow-up	^[24]^
4	20	M	PE, DVT, inherited AT deficiency	15 mg twice daily for 3 wk and then 20 mg once daily	On follow-up 8 mo later, no bleeding or recurrent thrombosis while on rivaroxaban	^[25]^
5	23	M	DVT, AT deficiency	15 mg twice daily for a week and then 10 mg twice daily for 7 mo	Over the 5-year follow-up period, no recurrent DVT was observed	^[26]^
6	24	F	PE, AT deficiency	15 mg twice daily for 21 d and switch to 20 mg once daily	On follow-up 3 mo later, no complications since initiation of therapy	^[27]^
7	28	M	DVT, PE, AT deficiency	20 mg per day	The thrombosis event did not happen anymore	^[28]^
8	28	F	VTE, AT deficiency	15 mg twice daily for 3 wk and then 20 mg once daily	Echocardiography after 10 d of treatment revealed complete resolution of the thrombus located in the inferior vena cava, while CTA revealed complete resolution of the PE. No recurrence of PE or DVT was found in 1-year follow-up	^[29]^
9	36	F	Multiple VTE, congenital AT deficiency, mutation in SERPINC	15 mg twice daily	Three days after rivaroxaban administrated, and before discharge, the thrombus remained stable. The d-dimer level decreased to 1002 ng/mL, and AT activity was 108%. The patient’s condition improved	^[30]^
10	41	M	Heterozygous Factor V Leiden mutation, acute thrombosis, drug-induced AT deficiency	15 mg twice daily for 13 d	AT levels returned to normal and clinical signs of the thrombosis resolved within 13 d	^[31]^
11	63	M	PE, AT deficiency	30 mg per day for 3 wk and then reduced to 15 mg per day	Four months later, a 12-lead electrocardiogram appeared normal and contrast-enhanced CT showed almost complete disappearance of the DVT and PE. The consolidation of left upper lobe completely disappeared	^[32]^

AT = antithrombin, CT = computed tomography, CTA = computed tomographic angiography, DVT = deep vein thrombosis, PE = pulmonary embolism, VTE = venous thromboembolism.

This case report may raise significant clinical considerations regarding the efficacy and safety of rivaroxaban in patents with AT deficiency, especially in terms of dosage and duration in children. The patient in this case, a 15-year-old senior high school student weighting 81.1 kg, was admitted with severe thrombosis progression, necessitating higher heparin doses (~72,000 U/d) to achieve the target aPTT. Subsequently, markedly low AT activity (27%) was detected, and it is known that heparin can decrease AT activity levels up to 30%, presumably by causing increased clearance of AT.^[34]^ This patient was diagnosed with AT deficiency after receiving high-dose heparin. As shown in Figure 1, after discontinuing heparin and switching to rivaroxaban, the value of AT was measured twice in 2 weeks and results showed that although it was still below the normal value, there was a gradually increasing trend. Therefore, it was difficult to determine whether the AT deficiency was congenital or acquired (caused by AT consumption following heparin use). Considering no plasma-derived AT or human recombinant AT supplementations available in our center, we changed the anticoagulant regimen to oral rivaroxaban 20 mg per day according to the EINSTEIN-Jr trials^[14]^ and the drug instructions. However, after 1 week of treatment with this standard regimen, the patient’s serum d-dimer level still elevated, which suggested the antithrombotic therapy may be insufficient. Consequently, the dose of rivaroxaban was increased to the acute thrombotic dose in adults (15 mg twice a day for 3 weeks, followed by a reduced dose of 20 mg per day), which was different from the EINSTEIN-Jr^[14]^ trials and was an off-label use of rivaroxaban. Monitoring of the serum d-dimer levels revealed a subsequent decrease, and the patient did not experience adverse events such as bleeding. On follow-up 6 months after discharge, a repeat d-dimer test showed a normal value (0.15 mg/L FEU) and repeat computed tomography showed that the thrombotic load was much less than before and no other side effects or complications occurred during the treatment, which proved the efficacy and safety of rivaroxaban in this adolescent case with VTE and AT deficiency.

## 4. Conclusions

This case not only contributes additional data on the efficacy and safety of direct oral factor Xa inhibitors in patients with VTE accompanied by AT deficiency, but also suggests that for Asian children patients with AT deficiency in the acute thrombotic phase, considering a higher dose of rivaroxaban (the dose of adult acute thrombotic phase) may be beneficial, especially when the children or teenager’s height, weight, and age are close to adults. Considering that the effective dose of rivaroxaban used by Asians is smaller than that of other ethnic groups, the dose used in this case was quite large. Consequently, rivaroxaban remains a viable anticoagulant option for patients with AT deficiency. However, further studies with larger populations are needed to validate the effectiveness and safety of rivaroxaban in this specific patient population.

## Author contributions

**Conceptualization:** Fang Liu, Li-Ping Du.

**Funding acquisition:** Chun-Su Liang, Li-Ping Du.

**Writing – review & editing:** Chun-Su Liang, Yue-Xin Chen, Yue-Dong Yue, Li-Ping Du.

**Writing – original draft:** Fang Liu.
